# Political representation of medical doctors in Switzerland’s executive and legislative branches in 2023

**DOI:** 10.12688/f1000research.130986.1

**Published:** 2023-02-27

**Authors:** Alexander Smith, Anna Buadze, Petra Stute, Michael Liebrenz

**Affiliations:** 1 Department of Forensic Psychiatry, University of Bern, Bern, 3012, Switzerland; 2 Department of Psychiatry, Psychotherapy and Psychosomatics, Psychiatric Hospital, University of Zurich, Zurich, Switzerland; 3 Department of Obstetrics and Gynecology, Inselspital, University Hospital Bern, Bern, Switzerland

**Keywords:** Healthcare policy, Physicians and politics, Political representation, Medical societies, Switzerland

## Abstract

**Background:** Healthcare policy is an important societal concern in Switzerland, often dominating the national agenda. In other countries, studies have explored the influence of physicians in public office on healthcare policies, but little is known about the representation of medical doctors in Switzerland's political structures, despite ongoing health-related debates.

**Methods**: In January 2023, we examined the proportion of registered doctors currently serving in Swiss governmental branches: the executive (the Federal Council) and the legislative (the Council of States and the National Council, together the United Federal Assembly). We used publicly available information to demarcate Federal, State, and National Councillors with professional medical backgrounds. We subsequently verified physician registrations using the Federal Office of Public Health’s “Register of Medical Professionals” (MedReg)

**Results**: Six physicians registered in MedReg were identified across the Federal Council and the United Federal Assembly in 2023, equivalent to 2.37% of the total number of Councillors in these chambers. This corresponds to 14.20% of members in the Federal Council (the executive chamber) and 2.03% of members in the United Federal Assembly (the legislative chamber).

**Conclusions**: Rates of physicians sitting in Switzerland’s Federal Council and United Federal Assembly are higher than general population trends for doctors per person. Nonetheless, physicians in Swiss legislative positions are proportionally lower than comparative data from the United States. We highlight how existing professional frameworks may already ensure medical doctors are sufficiently participating in Swiss healthcare debates outside of formal roles. We also suggest that more international evidence is needed to determine the benefits of physicians serving in public office.

## Introduction

The COVID-19 pandemic and rising living costs have reanimated socioeconomic concerns about health policies in Switzerland, which regularly capture the national agenda. ^1^ Owing to an ageing population and growing technological advancements, Swiss health insurance premiums were set to increase substantially in 2022, ^2^ with possible consequences for long-term fiscal sustainability. ^3^ Additional factors like pharmaceutical prices ^4^ and procedural backlogs ^2^ are exacerbating this situation. Recently, politicians have attempted to address these circumstances. For instance, the Swiss political party, The Centre, have suggested a federal vote on cost control measures and different lawmakers have recommended alternative initiatives. ^1^^,^^5^ However, at the time of writing, no consensus exists around potential interventions, ^1^ and historical proposals to modify the Swiss health system have not passed. ^6^

In other countries where healthcare is an important national issue, notably the United States, researchers have explored the role of physicians in governmental proceedings. In clinical subdisciplines recurrently exposed to legal matters, like forensic psychiatry, Piel has discussed expert advocacy. ^7^ Separately, professional organisations and medical practitioners frequently engage in lobbying activities. ^8^^,^^9^ In more formalised frameworks, physicians in political positions have historically attracted much inquiry, with papers dedicated to this issue from over fifty ^10^^,^^11^ and one hundred years ago. ^12^ For example, Oberstar has outlined various cases of US doctors occupying legislative seats and their influence on health policy. ^13^ For Kraus and Suarez, physician lawmakers and first-hand medical insights could be useful within political domains, particularly as research funding and care delivery can often be legislatively determined. ^9^ Moreover, anecdotally, Australian doctors have advocated for better representation across public office. ^14^

Previous work has examined the prevalence of medical doctors serving in state and federal legislative branches in the United States [e.g., Refs. 9, 15, 16]. Others have focussed on German physician-trained health ministers, ^17^ doctors in the United Kingdom’s parliament, ^18^ and biographical investigations into the medical background of various national leaders. ^19^ Nonetheless, to the authors’ knowledge, despite these studies in different national settings and ongoing Swiss healthcare policy concerns, there is scant awareness about registered physicians fulfilling formal political roles in Switzerland. Whilst Swiss media outlets accentuated the clinical expertise of the 2022 Federal President, Ignazio Cassis, during the COVID-19 pandemic, ^20^ there is limited scholarly evidence about the holistic representation of medical doctors in national governmental spheres.

Using secondary biographical information, we sought to identify the rates of registered physicians serving in Switzerland’s national executive branch (the Federal Council) and the legislative branches (the Council of States and the National Council, together the United Federal Assembly) in 2023. As Switzerland’s governmental tradition is distinctive for individuals holding jobs outside of politics, ^21^ our hypothesis was that the rates of serving physician-politicians would be higher than other geographical settings.

## Methods

In January 2023, we analysed publicly available information from the Swiss government and parliamentary websites, which list biographical records for executive and legislative positions: Federal Councillors, State Councillors and National Councillors (n=253). ^22^^–^^24^ Through these resources, we were able to gather vocational data for n=206 national politicians, with no specific filters or access restrictions. However, for certain State and National Councillors, secondary professional data were not displayed by these parliamentary sources. In these cases (n=47), two authors collected occupational details separately through an internet search of multiple sources, including the official websites of these Councillors and media reports. Full data for n=253 Councillors were then exported into a Microsoft Excel file and from this total sample, we demarcated politicians with relevant medical backgrounds.

Subsequently, we cross-referenced the information we collected by searching the “Register of Medical Professionals” (MedReg), which is managed by the Swiss Federal Office of Public Health. ^25^ This enabled us to validate the biographical data against registrations as medical physicians in MedReg, and ascertain age, clinical specialties, and gender; specifically, the latter was determined based on information presented in MedReg. Following this process of cross-comparison and validation, descriptive statistics were calculated using Microsoft Excel, which are displayed in our results.

This investigation did not entail primary research involving human participation or confidential data and thus approval from an institutional review board and informed consent were not sought. Furthermore, our study was based on publicly available web resources managed by third parties, with no access requirements or permissions. This followed existing protocols used by recently published research articles that identified physician-politicians in different geographical domains [e.g., Refs. 15, 16].

## Results

Our results are summarised in Table 1, which shows the total number of Federal Councillors, State Councillors, and National Councillors in 2023, alongside those who are registered doctors in MedReg.

**Table 1.** Registered physicians serving in Switzerland’s Federal Council, the State Council, and the National Councillors in 2023.

Governmental Body	Number of Councillors - 2023	Number of Councillors who are registered physicians in Switzerland per MedReg - 2023
Federal Council	7	1
State Council	46	1
National Council	200	4
Total	253	6

We found that 2.37% (n=6) of the total number of Councillors (n=253) across the executive and legislative domains of Swiss national politics were registered physicians in MedReg. This corresponds to registered medical doctors representing 14.20% of members in the Federal Council, 2.17% in the Council of States, and 2.00% in the National Council in 2023; or per each governmental branch, 14.20% of Switzerland’s national executive body and 2.03% of the national legislative body. These six physicians consisted of two females (33.33%) and four males (66.66%). Ages ranged from 45-49 (16.66%), 55-59 (25%), 60-64 (16.66%) and 65-69 (25%). In respect of their medical field of expertise, three were board-certified specialists in general internal medicine (50.00%), one was board-certified in general internal medicine and prevention and public health (16.66%), one was an expert in general internal medicine and endocrinology (16.66%), and one was board-certified as an ophthalmologist (16.66%).

## Discussion

Whilst rates of registered physicians serving in national political positions may appear insignificant, they are higher than demographic trends for doctors per person in Switzerland, ^26^ suggesting an overrepresentation compared to this general population-based metric. This is notable since practising physician numbers may be declining nationwide per previous studies. ^27^ Nevertheless, the number of doctors in the Swiss federal legislature is proportionally lower than contemporaneous results in other countries; in 2022, 3.10% of federal legislators in the US had professional backgrounds as physicians, ^15^ as compared to our finding of 2.03% in Switzerland’s United Federal Assembly in 2023. Using gender-based figures from the Swiss Medical Association (FMH), 44.90% of registered doctors in Switzerland are female, ^28^ indicating that they might be underrepresented in national public office per our study. Likewise, this could be apposite for medical subspecialties other than general internal medicine based on occupational statistics from the FMH. ^28^

In the authors’ opinion, broader political representation of doctors in Switzerland may prove advantageous for civil society and professional medical associations. ^13^ This could be especially timely given political and socioeconomic debates surrounding Swiss healthcare policies [e.g., Refs. 1, 2]. Significantly, medical expenditure was estimated to account for 11.80% of the country’s gross domestic product in 2020 ^29^ and a recent survey of 26,298 Swiss residents identified healthcare as a preeminent political concern. ^30^ Amidst these contexts, physicians could raise awareness about health-related issues and influence relevant policies as elected officials. For doctors, this could include discussions around insurance, research funding provisions, and patient care, where first-hand vocational experience of these notions has been beneficial in separate settings. ^9^^,^^13^ Further, physicians can be particularly valuable in high office positions; for instance, evidence shows that medically trained German health ministers improved hospital capacities, capital, and health insurance funding. ^17^

Nonetheless, the extensive coverage of medical societies and professional groups in Switzerland might mean that doctors are engaging at a political level outside of formal positions. Such organisations regularly provide policy guidance and undertake advocacy activities; notably, the FMH has a resource devoted to political matters. ^31^ Additionally, Switzerland’s distinctive consultation process of law-making ( *Vernehmlassung*) aims to involve stakeholders in legislative procedures ^32^ and may therefore already encompass physicians’ perspectives. Here, again, associations periodically facilitate responses to consultative requests around legislative considerations; for example, the FMH publicly declares its positions, ^33^ as does the Swiss Society of General Medicine, ^34^ amongst others. Correspondingly, participation within Federal Commissions can also facilitate dialogues between doctors and governmental administrations. These extra-parliamentary institutions are founded to provide advice on specific topics and are often dedicated to specialised medical areas [e.g., Refs. 35, 36].

### Limitations and directions for future research

We deemed our study to be the best available method to explore current levels of registered physicians in Switzerland’s executive and legislative political bodies; yet our approach has its limitations. Firstly, although other investigations into physicians in US governmental structures have adopted similar methodologies, ^15^^,^^16^ secondary analysis can invoke data availability and reproducibility questions. ^37^ For example, during data gathering, occupational information was not available on governmental or parliamentary websites for certain State (n=15) and National Councillors (n=32), and a supplementary internet search was required. This had the potential to introduce discrepancies; to mitigate against this, two authors consulted multiple internet sources, including official websites and press reports, to corroborate biographical information and the MedReg database was screened to validate physician registrations.

Moreover, we investigated Federal, State, and National Councillors in 2023 and not longitudinal trends. Due to data availability constraints, we deemed this appropriate to present current insights amidst contemporaneous healthcare debates; a comparative follow-up study after the Swiss Federal Elections in October 2023 could allow for more insights. Nonetheless, samples over a broader timeframe would allow for historical comparisons. Our work only captured physicians registered in MedReg and did not include other health-related professions or qualifications. Future research could encompass additional stakeholders involved in healthcare policy, such as nurses or insurance experts, and cantonal-level politicians for more exhaustive evaluations.

More generally, the extent to which physicians feel they are sufficiently participating in the governmental process through multiple professional mechanisms could form the basis for additional investigations. Whilst our study presents quantitative data about physicians in formal federal roles, qualitative findings about the wider political representation of Swiss physicians could better contextualise their influence on civil and health-related issues. This could be further complimented by extensive research in different countries and about how doctors serving in other national political structures can influence health policy debates.

## Conclusion

Healthcare policy is a substantial socio-political and economic concern in Switzerland, frequently capturing the national agenda. However, there is limited knowledge about the number of registered physicians serving in the Swiss Federal, State, and National Councils, despite similar studies in other countries.

Based on our findings, national political representation of medical doctors was above populational trends in Switzerland. Nevertheless, the number of physicians serving in the Swiss legislature (the United Federal Assembly) was lower than in the United States’ federal legislature, where comparable investigations have occurred. Further, females and clinical subspecialties other than general internal medicine may be professionally underrepresented in formal political roles.

Detailed work is needed to understand the implications of medical doctors occupying political offices and their representative mechanisms outside of formal federal positions in Switzerland. Moreover, further investigations should be encouraged in different political systems internationally. In general, this may help determine whether more physicians should be encouraged to run for elected office.

## Data Availability

Publicly available biographical information is obtainable from the Swiss government and parliamentary websites, which list occupational records for executive and legislative positions. Information about physician registrations is available by searching The Register of Medical Professionals. Data used in this study are: The “The seven members of the Federal Council” dataset of 2023, available from The portal of the Swiss government. The “National Council Members A–Z” dataset of 2023, available from The Federal Assembly – The Swiss Parliament. The “Council Of States Members A–Z” dataset of 2023, available from The Federal Assembly – The Swiss Parliament. The “Register of Medical Professionals” dataset of 2023, available from the Swiss Federal Office of Public Health.
