# Supplementary material for: Political representation of medical doctors in Switzerland’s executive and legislative branches in 2023
Source: F1000Res. 2023 Jul 20;12:219. Originally published 2023 Feb 27. [Version 3] doi: 10.12688/f1000research.130986.3 (PMC10336360; doi:10.12688/f1000research.130986.3)
Supplement: Peer review history

# Peer review: Reviewer response for version 2

**DOI:** 10.5256/f1000research.147317.r185606

**Published:** 2023-07-11

**Authors:** Michael J. Deml

**Affiliations:** 1 Department of Sociology, Institute of Sociological Research, Universite de Geneve, Geneva, Geneva, Switzerland

This was a well-written and clear investigation into the prevalence of registered physicians who hold political positions within the Swiss context. I only have a minor comment concerning the contextualization of health-system issues being on the research agenda in Switzerland, which was a result of the political will to finance the National Research Programme 74 "Smarter Health Care" through the Swiss National Science Foundation. It might be helpful for readers to know about this funding mechanism that funded 34 projects from 2017-2023 (my time period may not be fully accurate). 

 If the authors are interested, I would suggest consulting the synthesis reports from NRP74: https://www.nfp74.ch/en/WW3T81gCZ6vTbLpX/page/findings for further information. 

 There was also a peer-reviewed review conducted by myself and colleagues for the NRP74 steering committee. I am including it here out of potential interest to the authors and have no expectations whatsoever about this article being cited. 

https://www.ssph-journal.org/articles/10.3389/phrs.2022.1604434/full

 My suggestion would be to add 2-3 sentences about the political will for changes to the healthcare system in Switzerland through the funding of health-related research.

Is the work clearly and accurately presented and does it cite the current literature?

Partly

If applicable, is the statistical analysis and its interpretation appropriate?

Yes

Are all the source data underlying the results available to ensure full reproducibility?

Yes

Is the study design appropriate and is the work technically sound?

Yes

Are the conclusions drawn adequately supported by the results?

Yes

Are sufficient details of methods and analysis provided to allow replication by others?

Yes

Reviewer Expertise:

Health system research, vaccine hesitancy, health prevention, social sciences, infectious disease prevention

I confirm that I have read this submission and believe that I have an appropriate level of expertise to confirm that it is of an acceptable scientific standard.

## References

[1] : Megatrends in Healthcare: Review for the Swiss National Science Foundation's National Research Programme 74 (NRP74). *Public Health Rev* .2022;43: 10.3389/phrs.2022.1604434 1604434 10.3389/phrs.2022.1604434 35528712PMC9069234

# Response

**Published:** 2023-07-17

**Authors:** Alexander Smith

**Affiliations:** University of Bern, Switzerland

Thanks for taking the time to review our article and providing additional contextual information about the situation and health policy landscape in Switzerland. Per your comments and having read your very interesting paper, we have made reference to the Swiss National Science Foundation's scheme, Smarter Health Care. Our thanks again for your supportive comments, Dr Deml, and for taking the time to provide these insights.

# Peer review: Reviewer response for version 2

**DOI:** 10.5256/f1000research.147317.r171339

**Published:** 2023-05-02

**Authors:** Valerie Seror

**Affiliations:** 1 IRD, AP-HM, SSA, VITROME, Aix-Marseille Universite, Marseille, Provence-Alpes-Côte d'Azur, France

My comments had been addressed in this new version of the manuscript.

Is the work clearly and accurately presented and does it cite the current literature?

Yes

If applicable, is the statistical analysis and its interpretation appropriate?

Partly

Are all the source data underlying the results available to ensure full reproducibility?

Yes

Is the study design appropriate and is the work technically sound?

Partly

Are the conclusions drawn adequately supported by the results?

Partly

Are sufficient details of methods and analysis provided to allow replication by others?

Yes

Reviewer Expertise:

Health economics

I confirm that I have read this submission and believe that I have an appropriate level of expertise to confirm that it is of an acceptable scientific standard.

# Peer review: Reviewer response for version 1

**DOI:** 10.5256/f1000research.143788.r167296

**Published:** 2023-04-18

**Authors:** Valerie Seror

**Affiliations:** 1 IRD, AP-HM, SSA, VITROME, Aix-Marseille Universite, Marseille, Provence-Alpes-Côte d'Azur, France

This is a very well-written manuscript that explored the issue of the influence of physicians in political positions on health policy. As a first step, this study aimed at providing insight based on data gathered from publicly information accessible on the Internet without requirements or permissions. Data on age and gender of the members of the Federal, State and National Councils had been collected as well as data on the medical background of the physicians involved in these councils. Among a total number of 253 Councillors in 2023, 6 had been identified as medical doctors.

 Main comments:

1. Considering the relatively low number of physicians involved in the Councils, the authors pointed out a likely over-representation of the members of the Councils with medical background as compared to their rates in the general population. In order to get more insight on this issue, the authors could also question whether and to what extent occupational backgrounds of the members of the councils are representative of the Swiss population.
2. Considering the major issue of potential influence of physicians in political positions on health policy, the Discussion section could provide suggestions on whether to appraise such influence.

Is the work clearly and accurately presented and does it cite the current literature?

Yes

If applicable, is the statistical analysis and its interpretation appropriate?

Partly

Are all the source data underlying the results available to ensure full reproducibility?

Yes

Is the study design appropriate and is the work technically sound?

Partly

Are the conclusions drawn adequately supported by the results?

Partly

Are sufficient details of methods and analysis provided to allow replication by others?

Yes

Reviewer Expertise:

Health economics

I confirm that I have read this submission and believe that I have an appropriate level of expertise to confirm that it is of an acceptable scientific standard, however I have significant reservations, as outlined above.

# Response

**Published:** 2023-04-21

**Authors:** Alexander Smith

**Affiliations:** University of Bern, Switzerland

*Authors:* Dear Dr Seror, we would like to thank you for taking the time to review our paper and providing constructive and supportive feedback. We very much appreciate your valuable insights and have responded to the feedback you raised point-by-point below. We believe that these suggestions now strengthen the paper by providing additional contextualisation and reinforcing the theoretical basis for future international studies.

*Reviewer*: Considering the relatively low number of physicians involved in the Councils, the authors pointed out a likely over-representation of the members of the Councils with medical background as compared to their rates in the general population. In order to get more insight on this issue, the authors could also question whether and to what extent occupational backgrounds of the members of the councils are representative of the Swiss population.

*Authors*: This is an excellent point, thank you for raising this. In paragraph 1 of the discussion, we have now added other occupational comparisons (namely to farmers and lawyers from sources showing national trends) to better frame the representation of doctors in a national context.

*Reviewer: *Considering the major issue of potential influence of physicians in political positions on health policy, the Discussion section could provide suggestions on whether to appraise such influence.

*Authors*: Thank you for this suggestion, which will strengthen the basis for further studies. At the end of paragraph 2 of the discussion, we have included information about how to assess the political influence of doctors from a related study, alongside some suggestions from the political science literature. We have also acknowledged this at the end of the “Limitations and directions for future research section”. Thank you again for this important point.
